# Dietary salt reduction in rural patients with albuminurea using family and community support: the Mima study

**DOI:** 10.1186/1447-056X-9-6

**Published:** 2010-02-25

**Authors:** Shinji Fujiwara, Kazuhiko Kotani, Phillip J Brantley, Kokoro Tsuzaki, Yukiyo Matsuoka, Masayuki Domichi, Yoshiko Sano, Eiji Kajii, Naoki Sakane

**Affiliations:** 1 Mima City National Health Insurance Koyadaira Clinic. 295 Kawai, Koyadaira, Mima-shi, Tokushima 777-0302, Japan; 2 Division of Community and Family Medicine, Center for Community Medicine, Jichi Medical University. 3311-1 Yakushiji, Shimotsuke-shi, Tochigi 329-0498, Japan; 3 Division of Preventive Medicine, Clinical Research Institute, National Hospital Organization Kyoto Medical Center. 1-1 Mukuihata-cho, Fushimi-ku, Kyoto 612-8555, Japan; 4 Department of Clinical Laboratory Medicine, Jichi Medical University. 3311-1 Yakushiji, Shimotsuke-shi, Tochigi 329-0498, Japan; 5 Division of Public Health, Center for Community Medicine, Jichi Medical University. 3311-1 Yakushiji, Shimotsuke-shi, Tochigi 329-0498, Japan; 6 Department of Behavioral Medicine, Pennington Biomedical Research Center. 6400 Perkins Road, Baton Rouge, Louisiana 70808, USA

## Abstract

**Background:**

Residents of rural communities are often more socially connected compared to urban dwellers. Using family and community support to motivate health behavior change may be useful in rural settings. The objective of this study was to pilot a salt reduction (SR) intervention for rural albuminuria patients using support from family and neighborhood residents compared to a usual care condition. The primary outcome was change in urine albumin-creatinine ratio (ACR).

**Methods:**

All consecutive outpatients with an ACR >= 30 mg/gCr were recruited from the Koyadaira Clinic. Patients self-selected their participation in the intervention group (IG) or the control group (CG) because the rural population expressed concern about not being treated at the same time. In the IG, patients and their families were educated in SR for 30 minutes in their home by experienced dieticians. In addition, patients, families and neighborhood residents were also educated in SR for 2 hours at a public town meeting hall, with educational content encouraging reduction in salt intake through interactive activity. The CG received conventional treatment, and ACR and blood pressure (BP) were measured after 3 months.

**Results:**

Of the 37 subjects recruited (20 male, 16 female, mean age; 72.8 ± 9.2 years), 36 completed the 3-month follow up and were analyzed. In the IG, ACR decreased significantly from baseline (706 ± 1,081 to 440 ± 656; t = 2.28, p = 0.04) and was reduced compared to the CG (213 ± 323 to 164 ± 162; F = 3.50, p = 0.07), a treatment effect approaching significance. Systolic BP in the IG (145 ± 14 to 131 ± 13 mmHg; t = 3.83, p = 0.002) also decreased significantly compared to the CG (135 ± 13 to 131 ± 14; F = 4.40, p = 0.04).

**Conclusions:**

Simultaneous education of patients, their families and neighborhood residents may be important in rural areas for treatments and interventions requiring health behavior change.

**Trial registration:**

UMIN000001972

## Background

Albuminuria is a risk marker of renal failure (RF) and its reduction suggests a slowing of RF [1]. Salt reduction (SR) improves the urine albumin-to-creatinin ratio (ACR) directly and/or via a reduction of blood pressure (BP) [2].

The Koyadaira area is an isolated rural community of approximately 1,000 residents in the Mima City, and has the highest mortality associated with RF in Japan [3]. The local government has provided the population with regular community health promotion classes, some of which target SR, but with little success at improving RF outcomes. In contrast, a Japanese urban hospital practice showed that SR, guided individually by dieticians, decreased urinary protein excretion [4]. There is no such data available in rural communities, where the health status and health-related behaviors can differ substantially from that of urban communities [5]. Successive doctors at the only clinic in this area have tried to tackle this problem using individual dietary guidance, but without success. Patients, their families and neighborhood residents are typically more closely related with each other in rural areas compared to urban communities [6], suggesting a family and community approach to health behavior change may be warranted.

Mima City National Health Insurance Koyadaira Clinic (Koyadaira Clinic) provides care for almost half of all the positive albuminurea patients in this area. We conducted a pilot study with these patients to test the feasibility and effectiveness of a SR intervention that included patients' family members and neighbors to help motivate the patients to make dietary changes.

## Methods

### Study design

Non-randomized controlled trial.

### Subjects

All consecutive outpatients with albuminurea (ACR >= 30 mg/gCr) at Koyadaira Clinic from May to October 2006 were registered with this study which was approved by the Ethics Committee of the National Hospital Organization Kyoto Medical Center. Each subject gave written informed consent. Participants had no clinical features of RF, ischemic heart disease, or stroke. All were invited to join a health promotion class and invite some of their family and friends to accompany them. Those who attended the class made up the intervention group (IG). Those who chose not to attend the class made up the control group (CG).

### Intervention

Patients in the IG were educated during a 2-hour health promotion class with their family and neighbors at a public town meeting hall. In addition, a 30-minute session on dietary change was held with their families at their home. Participants' medical diagnoses were not disclosed in front of neighborhood residents to protect patients' privacy. Four dieticians from outside the community conducted the education sessions. They used interactive exercises, such as pair and small group discussion, quizzes to estimate the amount of salt in foods, etc., to encourage participants' reduced consumption of traditionally salty Japanese foods. Examples of high salt foods, eaten frequently by elderly people in Japan, are miso soup, pickled vegetables and soy sauce. The dieticians asked the participants to set goals of behavior change for reducing salt intake and to record their behavior in daily logs. One month later, the dieticians mailed all participants a reminder about their salt reduction goal.

The doctor, three nurses at the clinic, and two public health nurses in the area assisted with the intervention activities and followed the subjects for three months post intervention. They reviewed participants' food monitoring logs and provided encouragement for dietary change during monthly visits. The patients in the CG received usual care, which consisted of monthly visits and physician advice to reduce salt.

### Measures

The primary outcome was change in ACR measured before and after the 3 month intervention. ACR was determined by turbidimetric immunoassay in the early morning urine sample (N-assay TIA Micro Alb NITTOBO, Nittobo Medical Co. Ltd., Tokyo, Japan). Secondary outcomes were changes in systolic and diastolic blood pressures. The blood pressure was measured two times at ten-minute intervals in seated subjects using a mercury sphygmomanometer after 5 minutes at rest.

Serum creatinine (Cr) levels were determined enzymatically and blood urea nitrogen (BUN) was determined by the urease method. Estimated glomerular filtration rate (eGFR) (mL/min/1.73 m^2^) was calculated using the eGFR equation for Japanese, eGFR = 194 × Age^-0.287 ^× Cr^-1.094 ^(×0.739, if female) [7]. After an overnight fast, body weight was measured using a body fat analyzer (HBS-354-W, OMRON, Kyoto, Japan); and body mass index (BMI) was calculated as weight in kilograms divided by height in meters squared.

The dieticians examined salt intake in the IG during the group and individual sessions, using Excel Eiyokun (version 4.5, KENPAKUSHA, Tokyo, Japan) for the assessment of dietary intake by food frequency questionnaire.

### Analyses

Statistical analyses were conducted using SPSS II for windows (version 11.01J, SPSS Inc., Chicago, IL, USA). All data are reported as the means ± SD or n (%). Baseline comparisons were performed with Mann-Whitney U-test and Chi-square test. A paired t-test was used to compare mean systolic and diastolic blood pressures and ACR before and after the 3 month intervention in each group. Two-way analysis of variance (ANOVA) was performed to compare the difference in changes of ACR, systolic and diastolic pressure in two groups. The determinant of statistical significance for all analyses was p < 0.05.

## Results

### Study participants

Of all consecutive 37 outpatients with albuminurea (20 male, 16 female, mean age; 72.8 ± 9.2 years, hypertension 94.6%, diabetes 37.8%) enrolled in this study, 36 completed the 3-month follow up. One subject in the IG dropped out on her own initiative. Data of 14 patients in the IG and 22 patients in the CG were analyzed.

Baseline characteristics of the sample are shown in Table 1. The proportion of males and the levels of systolic blood pressure and Hb_A1C _in the IG were significantly higher than that in the CG. Salt intake was estimated only in the IG and found to be 12.0 ± 3.5 g/day at baseline.

**Table 1:** Baseline characteristics

Variables	Intervention group (n = 14)	Control group (n = 22)	P value^a^
Sex (male/female)	11/3	9/13	0.041
Age (y)	69.0 ± 11.0	75.1 ± 7.2	0.111
Body Mass Index (kg/m^2^)	24.1 ± 3.1	24.9 ± 3.8	0.417
Systolic blood pressure (mmHg)	145.1 ± 13.9	134.9 ± 13.1	0.041
Diastolic blood pressure (mmHg)	67.1 ± 8.3	66.4 ± 12.2	0.733
Hb_A1C _(%)	6.7 ± 1.8	5.1 ± 0.4	<0.001
BUN (mg/dL)	15.2 ± 3.3	18.9 ± 6.6	0.149
Cr (mg/dL)	0.8 ± 0.2	1.0 ± 0.4	0.745
ACR (mg/gCr)	706.1 ± 1082.1	212.5 ± 322.5	0.427
eGFR^b ^(mL/min/1.73 m^2^)	71.6 ± 23.4	59.6 ± 23.3	0.173
Stage of chronic kidney disease			
Stage 1: eGFR^b ^>= 90	2 (14.3)	3 (13.6)	
Stage 2: 60~89	7 (50.0)	10 (45.5)	
Stage 3: 30~59	5 (35.7)	5 (22.7)	
Stage 4: 15~29	0 (0.0)	4 (18.2)	
Stage 5: <15	0 (0.0)	0 (0.0)	0.376
Antihypertensive drugs			
Any antihypertensive drugs	11 (78.6)	19 (86.4)	0.541
Rennin-angiotensin system blocking drugs	11 (78.6)	15 (68.2)	0.497
Dietary salt intake^c ^(g/day)	12.0 ± 3.5	-	-

Abbreviation: Hb_A1C_, glycosylated hemoglobin; BUN, blood urea nitrogen; Cr, serum creatinine; ACR, urine albumin-creatinine ratio; eGFR, estimated glomerular filtration rate

Data are mean ± SD or n (%).

^a^P value were calculated by Mann-Whitney U-test and Chi-square test if categorical variables were used.

^b^Calculated using eGFR equation for Japanese, eGFR = 194 × Cr^-1.094 ^× Age^-0.287 ^(× 0.739, if female) [7].

^c^Only subjects in intervention group were examined by dieticians using Food Frequency Questionnaire.

### Changes in ACR and blood pressure

Primary and secondary outcomes at three months are shown in Table 2. ACR decreased significantly in the IG, and approached significance compared to that of the CG (p = 0.070). Systolic blood pressure decreased significantly in both the within and between-group comparisons.

**Table 2:** Primary and secondary outcome at 3 months

Variables	Intervention group (n = 14)	Control group (n = 22)	Between-group difference (p value^a^)
**Primary outcome**			
ACR (mg/gCr)			
Baseline	706.1 ± 1081.2	212.5 ± 322.5	
After 3 months	440.0 ± 656.3	163.5 ± 161.5	
Change	-266.1 ± 436.3	-49.0 ± 261.2	0.070
Within-group difference (p value^b^)	0.040	0.388	

**Secondary outcome**			
Systolic blood pressure (mmHg)			
Baseline	145.1 ± 13.9	134.9 ± 13.1	
After 3 months	130.9 ± 12.9	130.9 ± 14.0	
Change	-14.3 ± 13.9	-4.0 ± 14.6	0.043
Within-group difference (p value^b^)	0.002	0.212	
Diastolic blood pressure (mmHg)			
Baseline	67.1 ± 8.3	66.4 ± 12.2	
After 3 months	62.7 ± 7.6	66.8 ± 7.8	
Change	-4.4 ± 7.6	0.5 ± 11.3	0.165
Within-group difference (p value^b^)	0.048	0.853	

Abbreviation: ACR, urine albumin-creatinine ratio

Data are mean ± SD.

^a^Two-way ANOVA

^b^Paired t-test

## Discussion

The present study shows promise for decreasing ACR in the group of albuminurea patients encouraged to reduce salt intake with their families and neighborhood residents together. The intervention focused on goal setting for dietary SR and used family and neighbors to offer support to patients in a rural area where close human relationships remain. The study shows innovation by including patients' families and neighborhood residents in activities designed to encourage reduction of the patients' dietary salt intake. To our knowledge, this is the first report on social support to involve patients' neighbors in a patient education intervention.

It is meaningful for the subjects to be educated in sodium reduction. Salt intake in the IG at baseline was 12.0 g/day, which is more than the 11.2 g/day, mean of the Japanese population [8]. Recommended salt intake is less than 10 g/day for the general population [9] and less than 6 g/day for chronic kidney disease patients [7].

This study was conducted as a pilot study in a rural population sensitive to not being treated at the same time, therefore, we let the subjects decide whether to join the intervention activities or not. As a result, characteristics were different between the IG and the CG. Randomized controlled trials, likely requiring multiple centers, are needed in the future.

A major limitation of the present study relates to sample size. Convenience sampling was used, and no sample size determination was performed. Therefore it is possible that the statistically insignificant findings are the result of low power.

In addition, there was possible treatment contamination (sharing of intervention information) between the IG and the CG. The study community is so small that subjects in both groups may have shared the content of the intervention activities when chatting in their neighborhood, at the waiting room at Koyadaira Clinic, etc. Since outcomes tended to improve in both groups, one might assume that both groups benefited from the intervention. Finally, we should have measured salt intake in the CG as was done for the IG. Baseline characteristics were different between the IG and the CG, so it is possible that salt intake was not similar between the two groups.

## Conclusions

In addition to the patients themselves, simultaneous education of families and neighborhood residents may improve outcomes in rural patients, suggesting a possible community element to motivation for health behavior change in this population. Future studies are needed to examine this hypothesis.

## Competing interests

The authors declare that they have no competing interests.

## Authors' contributions

SF conceptualized, designed, acquired funding, collected and analyzed data, and drafted the manuscript. KK, EK and NS contributed to conception, design, analysis and interpretation of data and writing the manuscript. PJB participated in revising the manuscript critically for interpretation of data and meaning of this study design. PJB and EK gave final approval of the versions to be published. KT, YM, MD and YS managed this study, organized the intervention activities and collected data. All authors read and approved the final manuscript.
